# Can head sway patterns differentiate between patients with Meniere’s disease vs. peripheral vestibular hypofunction?

**DOI:** 10.3389/fneur.2024.1347335

**Published:** 2024-02-29

**Authors:** Jennifer L. Kelly, Maura Cosetti, Anat V. Lubetzky

**Affiliations:** ^1^Ear Institute, New York Eye and Ear Infirmary of Mount Sinai, New York, NY, United States; ^2^Department of Otolaryngology - Head and Neck Surgery, Icahn School of Medicine at Mount Sinai, New York, NY, United States; ^3^Department of Physical Therapy, Steinhardt School of Culture, Education and Human Development, New York University, New York, NY, United States

**Keywords:** Meniere’s disease, vestibular hypofunction, postural control, head kinematics, balance, vestibular disorders

## Abstract

**Background:**

Meniere’s disease (MD) is defined by episodic vertigo, unilateral sensorineural hearing loss and fluctuating aural symptoms. Due to the variable clinical presentation, objective tests of MD may have significant diagnostic utility. Head kinematics derived from a head-mounted display (HMD) have demonstrated to be sensitive to vestibular dysfunction. The purpose of this pilot study was to investigate whether head sway can differentiate between patients with MD, vestibular hypofunction (VH) and healthy controls.

**Materials/methods:**

80 adults (30 healthy controls, 32 with VH, and 18 with MD) were recruited from a tertiary vestibular clinic. All underwent a postural control assessment using the HTC Vive Pro Eye HMD that recorded head sway in the anterior–posterior (AP), medio-lateral (ML), pitch, yaw and roll direction. Participants were tested with 2 levels of visual load: a static versus oscillating star display. Each scene lasted 60 s and was repeated twice. Sway in each direction was quantified using root mean square velocity (VRMS) for the first 20 s and full 60 s of each scene.

**Results:**

Static visual: participants with VH showed significantly larger head VRMS than controls in the AP (60 s and 20 s) and pitch (20 s) directions. Dynamic visual: participants with VH showed significantly larger head VRMS than controls all directions for both the 60 and 20 s analysis. Participants with MD did not differ significantly from the control or the VH group.

**Conclusion:**

While limited in numbers, Patients with MD had a high variability in head sway in all directions, and their average head sway was between controls and those with VH. A larger sample as well as patients with worse symptoms at time of testing could elucidate whether head sway via HMD could become a viable test in this population. A similar finding between 20- and 60-s scene and the full portability of the system with an in-clinic testing setup could help these future endeavors. Head sway derived from HMD is sensitive to VH and can be clinically useful as an outcome measure to evaluate sensory integration for postural control.

## Introduction

Meniere’s disease (MD) is a clinical syndrome that presents with recurrent, spontaneous episodic vertigo lasting 20 min to 12 h, fluctuating unilateral sensorineural hearing loss (SNHL) and ipsilateral, fluctuating aural symptoms (fullness and tinnitus). Incidence of MD has been estimated to be between 13 to 190 per 100,000 people with the larger estimate reported in the US (1, 2). The current international classification system adopted by both the Barany Society and the American Academy of Otolaryngology—Head and Neck Surgery (AAO-HNS) has two categories of diagnosis—‘definite’ and ‘probable’ (1, 3) both of which require that the symptoms are “not more likely to be due to an alternative diagnosis.” Indeed, the differential diagnosis of MD is challenging as it can mimic a myriad of other episodic vestibular disorders, such as vestibular migraine, labyrinthitis and autoimmune inner ear disease (3, 4). Additionally, between episodes, both audiovestibular testing and clinical exam findings may be normal (2). Though audiometric patterns of low-to moderate frequency SNHL are cited in the international diagnostic criteria, clinical diagnosis relies heavily upon patient history to separate MD from its mimics (5). For these reasons, any objective marker of MD can be highly clinically useful and increase the opportunity for timely and appropriate medical management.

Unlike MD, which is a fluctuating condition, unilateral vestibular hypofunction (VH) is defined as reduced function of one of the peripheral vestibular sensory organs and/or vestibular nerves with characteristic symptoms of dizziness and imbalance (6). It has been estimated that VH affects between 53 and 95 million adults in Europe and the US (7). The annual incidence of VH varied by countries from 3.5 to 15.5 per 100,000 (8, 9). Imbalance, a common characteristic of VH, is not included in the diagnostic criteria of MD, even though it is a predominant complaint among these individuals. In a retrospective quality of life survey of 539 people with MD, 44.4% reported balance related problems, 58% reported hearing problems, 50% reported tinnitus and aural fullness, and 39.5% reported vertigo (10). Concordant with the high degree of clinical variability, complaints of imbalance and abnormalities of postural control in MD are poorly characterized, likely at least partially because of the episodic nature of the disorder. A handful of studies demonstrated greater dependence on visual and somatosensory information in maintaining an upright posture in people with MD compared with healthy controls (11–13) suggesting abnormalities of postural control may persist between episodes.

Prior research has suggested that head sway is sensitive to changes post-surgery (14) or rehabilitation (15) in vestibular disorders, and may assist in the differential diagnosis of vestibular disorders in the clinical setting (16–18). Specifically, prior studies have observed differences in head sway measured by Head Mounted Displays (HMD) between patients with vestibular hypofunction (VH), Persistent Postural Perceptual Dizziness (PPPD) and controls in response to visual perturbations. In patients with VH, head sway was increased as the balance task became more challenging (such as standing on foam) (15–17, 19, 20). In contrast, differences in head sway between patients with PPPD and healthy controls decreased with the increased challenge of the task (visual or cognitive) (21). The authors hypothesized that high levels of symptoms pre and post assessment or high anxiety explained participants’ reduction of head movement (“freezing”). These findings suggest that head sway may serve as an objective marker to differentiate between some vestibular disorders, however little is known about its application to MD.

The purpose of the current pilot study was to investigate whether head sway during an in-clinic, HMD-based postural control assessment can differentiate between patients with MD, unilateral peripheral VH and healthy controls. While individuals with VH have a known deficit in sensory integration for postural control (15, 21–23), MD is a fluctuating condition with inconsistent results on clinical exam. This may result in larger differences between VH and controls than between MD and controls. A secondary aim was to determine whether data acquired over 20 s is similar to results from a 60-s assessment. Historically, studies using similar HMD paradigms used 60–180 s scenes to allow for entrainment to the visual stimulus (24–26). However, clinical assessments of postural steadiness with visual changes (such as standing with eyes closed) typically measure balance for 20–30 s. If shorter conditions show similar findings, this will have important implications to the translation of this assessment and its feasibility in a clinical setting.

## Materials and methods

Participants with unilateral vestibular hypofunction (VH) and unilateral Meniere’s disease (MD) were recruited from a tertiary, urban, academic, otology practice and outpatient vestibular rehabilitation clinic at the Ear Institute at New York Eye and Ear Infirmary of Mount Sinai. Diagnosis of peripheral vestibular hypofunction was made by either bedside clinical exam from a physical therapist trained in vestibular rehabilitation (positive findings on bedside head impulse test, head shaking nystagmus or gaze evoked nystagmus consistent with peripheral loss) or from VNG results with caloric difference > 25% (8, 27). Diagnosis of definite Meniere’s disease was made by a neurotologist based upon current clinical practice guidelines (1, 3). Controls were recruited from community or academic hospital/university settings. This study was approved by both the Mount Sinai IRB 18–00431 and the NYU IRB-FY2016-155.

Participants were excluded if they had any neurological condition or orthopedic condition that could influence balance, if they used an assistive device for ambulation, or if they had an uncorrected vision impairment. All participants signed an informed consent form and passed a visual screen using the ETDRS to confirm normal or corrected to normal vision. Adequate vision was considered a visual acuity of 20/60 (the NYS acceptable standard for driving). They then completed the following validated metrics: the Dizziness Handicap Inventory (DHI), the Activities Specific Balance Confidence Scale (ABC), Visual Vertigo Analog Scale (VVAS), Symptom Sickness Questionnaire (SSQ; pre and post postural control assessment) and a demographic questionnaire. The VVAS is a subjective scale where the participants mark the intensity of their dizziness on a scale of 0 to 10 cm for each of 9 situations of visual motion that typically provoke dizziness (28). The score is calculated by measuring each item in centimeters, averaging the scores and multiplying by 10. Symptom severity can be classified as none (0), mild (0.1 to 40), moderate (40–70) or severe (above 70) (29, 30). The ABC is a validated subjective measure of a participant’s confidence in performing specific activities without falling. Each item is scored from 0% (no confidence in one’s balance) to 100% (full confidence in one’s balance) (31). A score of less than 67% indicates increased fall risk in community dwelling adults (32). The DHI is a well-recognized, 25 item validated questionnaire querying dizziness handicap across functional, emotional, and physical domains. Each item is scored as ‘no’, ‘sometimes’ or ‘yes’ to evaluate self-perceived disability imposed by dizziness (31, 33). The DHI is classified as mild (under 30), moderate (31–60) or severe (61–100) disability due to dizziness (34). The SSQ is a self-reported questionnaire given both before and after the session, which includes questions beginning with “are you experiencing any “and then different symptoms such as fatigue, general discomfort, blurred vision, dizziness etc. (35). Items are scored as “none” (0), “slight” (1), “moderate” (2) or “severe” (3). The participants then completed the following functional outcome measures: the Four Square Step Test (FSST) and the Timed up and Go test (TUG). The TUG is a standardized walking test to measure walking ability and fall risk, in which participants are instructed to rise up from a chair, walk at their comfortable speed for 10 feet, turn around a cone, walk back and sit down (36). We recorded the faster of two trials. The FSST is a multidirectional stepping test of dynamic balance and coordination. Participants are asked to step over 4 canes on the floor in a clockwise and then counterclockwise direction while being timed (37). Participants completed one practice trial and then we recorded the faster performance out of two trials. A result over 12 s on the TUG and over 15 s on the FSST reflects increased fall risk in community-dwelling older adults (34, 37).

**Figure 1:**
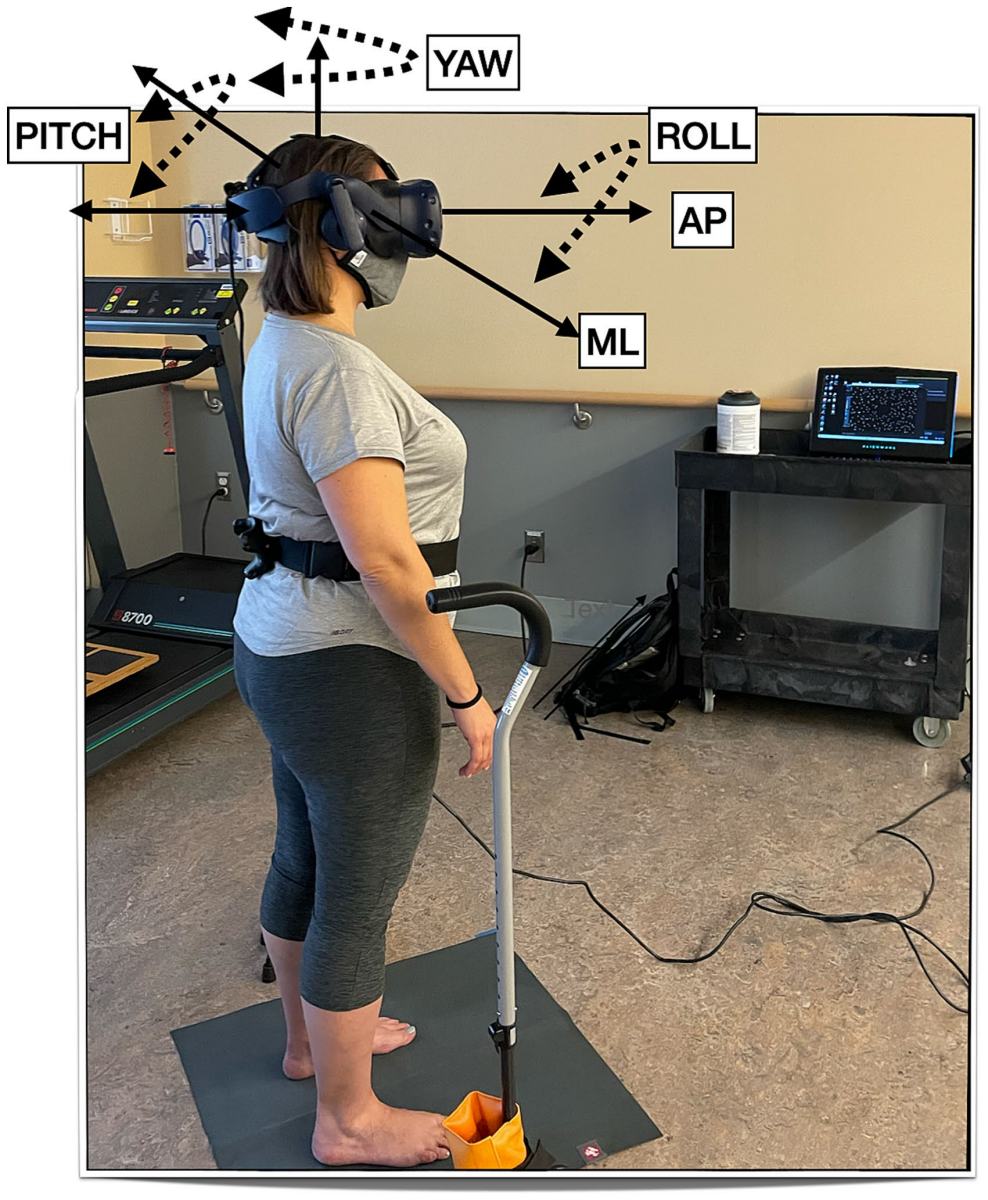
Experimental setup.

For the postural control assessment, participants stood on the floor barefoot, hips-width apart, and wore the HTC Vive Pro Eye (HTC, Taoyuan City, Taiwan) HMD (portable virtual reality headset) with the HMD’s built-in headphones. They were asked to look straight ahead and do whatever felt natural to them to maintain their balance. The well-established protocol consists of a visual surround 3-wall display of a ‘stars’ scene (38, 39). We then displayed 2 levels of visual load: static (the walls did not move) or dynamic (the walls were moving anterior–posterior (AP) at 0.2 Hz and 0.032 meters). They were guarded by either a licensed physical therapist or a physical therapy student during the postural control assessment. The entire session took 30 min. See Figure 1 for the experimental setup.

### Data processing

We used a custom-made Unity software written for the HTC Vive to record head sway data at 90 Hz and MATLAB R2023a (MathWorks, Natick, MA) to process and analyze data. We calculated head Root Mean Square Velocity (VRMS, cm/s or rad/s) in 5 directions (Anterior–posterior, Medio-lateral, Pitch, Yaw, Roll) (15): VRMS is the difference in position between two consecutive data points divided by the average time interval after a low-pass 4^th^ order Butterworth filter with a cutoff frequency at 10 Hz is applied (40). The velocity at each point is then squared and summed. The square root of the sum is then divided by the number of data points (41).

### Statistical analysis

We used descriptive statistics (mean, median, SD, min, max) for all variables (demographic, self-reported, functional and head sway). We used scatterplots and Pearson’s correlations to evaluate the relationship between demographics variables and head sway. For each of the datasets (60 s and 20 s), we used 10 Independent Sample Kruskal-Wallis Tests to evaluate the main effect of ‘group’ on VRMS for the static scene and the dynamic scene separately in 5 directions (AP, ML, Pitch, Yawl, Roll). If a significant main effect of ‘group’ was observed we conducted pairwise comparisons (control to VH, control to MD, VH to MD) with Dunn-Bonferroni corrections. To evaluate the effect of change in visual load on VRMS in each direction (AP, ML, Pitch, Yaw, Roll) within a group, we used Wilcoxon Signed Ranks Test. We also report a median, 25th (Q25) and 75th (Q75) percentile and Cohen’s D effect size (ES) for each significant main effect.

## Results

### Sample

**Figure 2:**
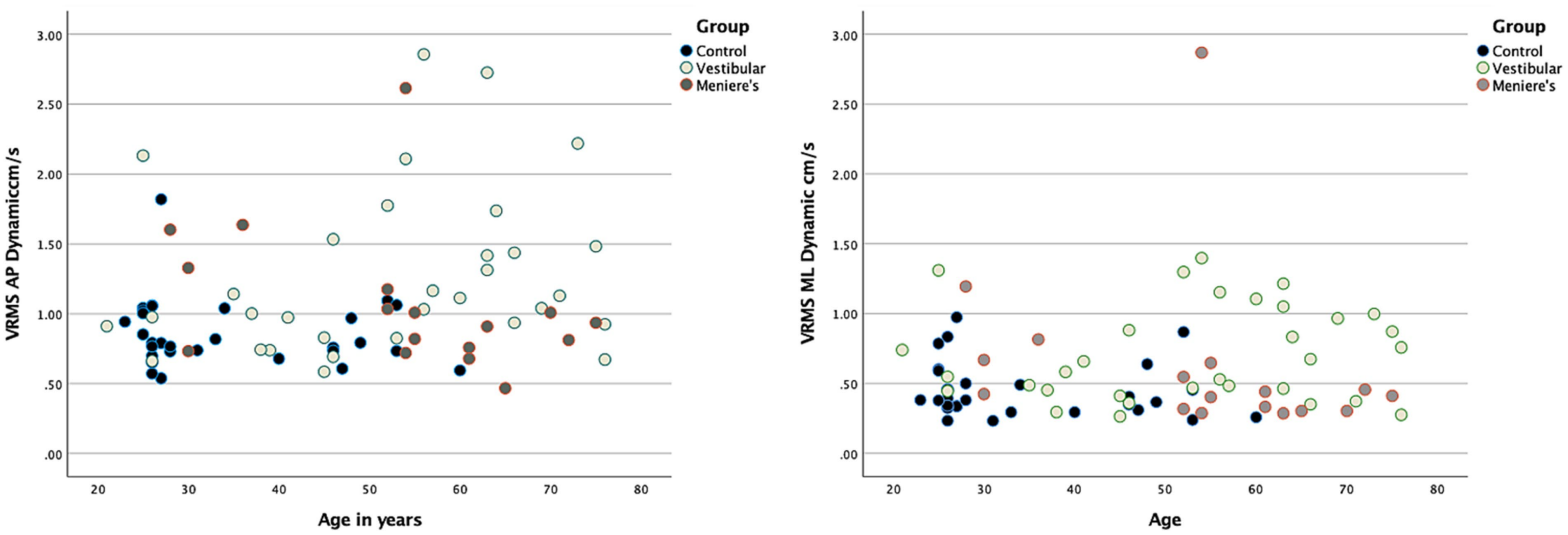
Scatterplots demonstrating no relationship between head VRMS and age for the three groups. A representative example is shown for 60 s AP (left-hand side) and ML (right-hand side).

80 adults (30 healthy controls, 32 with VH, and 18 with MD) were recruited (Table 1). Patients with VH had the following diagnoses: vestibular neuritis, acoustic neuroma, and labyrinthitis. Overall, the control group was younger whereas the VH and MD group were the same age on average. Average self-reported dizziness (VVAS, DHI) was a little higher for the VH group than the MD group, Participants with MD covered the entire range of the VVAS scale (from 0 to 100) and also had participants with 0 DHI. Functionally, the groups were similar on the TUG and FSST and below established cut-offs for fall risk (37, 42). None of the demographic variables, including age, correlated with head sway on any condition with all correlations ranging between 0.03 to 0.2 except for VVAS values that correlated at 0.4 with AP sway. Figure 2 shows AP and ML VRMS by age (correlations ranging from 0.026 to 0.174).

**Table 1:** Description of the sample.

Variable	Controls (*N* = 30)	Peripheral vestibular hypofunction (VH, *N* = 32)	Meniere’s disease (MD, *N* = 18)
Age	Mean = 34.47 Median = 27.5 SD = 11.37 Min = 23 Max = 60	Mean = 52.59 Median = 55.00 SD = 16.06 Min = 21 Max = 76	Mean = 53.40 Median = 54.50 SD = 14.37 Min = 28 Max = 75
Gender (N of females, %)	N = 16 53%	N = 17 53%	N = 4 22.2%
Weight (Kg)	Mean = 69.17 Median = 69.63 SD = 16.23 Min = 47.63 Max = 127.01	Mean = 73.82 Median = 74.16 SD = 14.20 Min = 45.36 Max = 102.06	Mean = 87.40 Median = 90.27 SD = 19.27 Min = 46.72 Max = 120.20
Height (cm)	Mean = 168.52 Median = 167.64 SD = 10.63 Min = 149.86 Max = 195.58	Mean = 167.05 Median = 166.37 SD = 10.00 Min = 149.86 Max = 187.96	Mean = 173.43 Median = 176.53 SD = 11.94 Min = 147.32 Max = 195.58
DHI	Mean = 0.13 Median = 0.00 SD = 0.73 Min = 0 Max = 4	Mean = 48.19 Median = 47.00 SD = 23.60 Min = 4 Max = 100	Mean = 34.11 Median = 25.00 SD = 30.44 Min = 0 Max = 82
ABC (%)	Mean = 98.79 Median = 99.19 SD = 1.24 Min = 96.25 Max = 100	Mean = 71.24 Median = 74.38 SD = 21.02 Min = 8.13 Max = 100	Mean = 82.21 Median = 90.00 SD = 22.60 Min = 20.00 Max = 99.00
VVAS	Mean = 1.56 Median = 0.00 SD = 3.45 Min = 0.00 Max = 15.78	Mean = 34.38 Median = 31.11 SD = 23.72 Min = 0.78 Max = 88.89	Mean = 22.48 Median = 12.28 SD = 25.10 Min = 0.00 Max = 100.00
TUG (sec)	Mean = 6.19 Median = 6.36 SD = 1.14 Min = 3.24 Max = 8.65	Mean = 8.16 Median = 8.20 SD = 2.14 Min = 4.72 Max = 14.56	Mean = 8.40 Median = 7.99 SD = 1.77 Min = 5.91 Max = 13.40
FSST (sec)	Mean = 6.69 Median = 6.53 SD = 2.23 Min = 3.18 Max = 14.92	Mean = 10.71 Median = 10.41 SD = 3.54 Min = 5.07 Max = 21.60	Mean = 11.02 Median = 9.64 SD = 4.71 Min = 6.12 Max = 25.05
SSQ pre	Mean = 0.20 Median = 0.00 SD = 0.61 Min = 0 Max = 3	Mean = 3.97 Median = 3.00 SD = 3.38 Min = 0 Max = 10	Mean = 2.72 Median = 1.00 SD = 4.13 Min = 0 Max = 16
SSQ post	Mean = 0.23 Median = 0.00 SD = 0.68 Min = 0 Max = 3	Mean = 4.16 Median = 4.00 SD = 4.35 Min = 0 Max = 14	Mean = 2.78 Median = 1.00 SD = 5.38 Min = 0 Max = 20

### 60 s analysis

**Figure 3:**
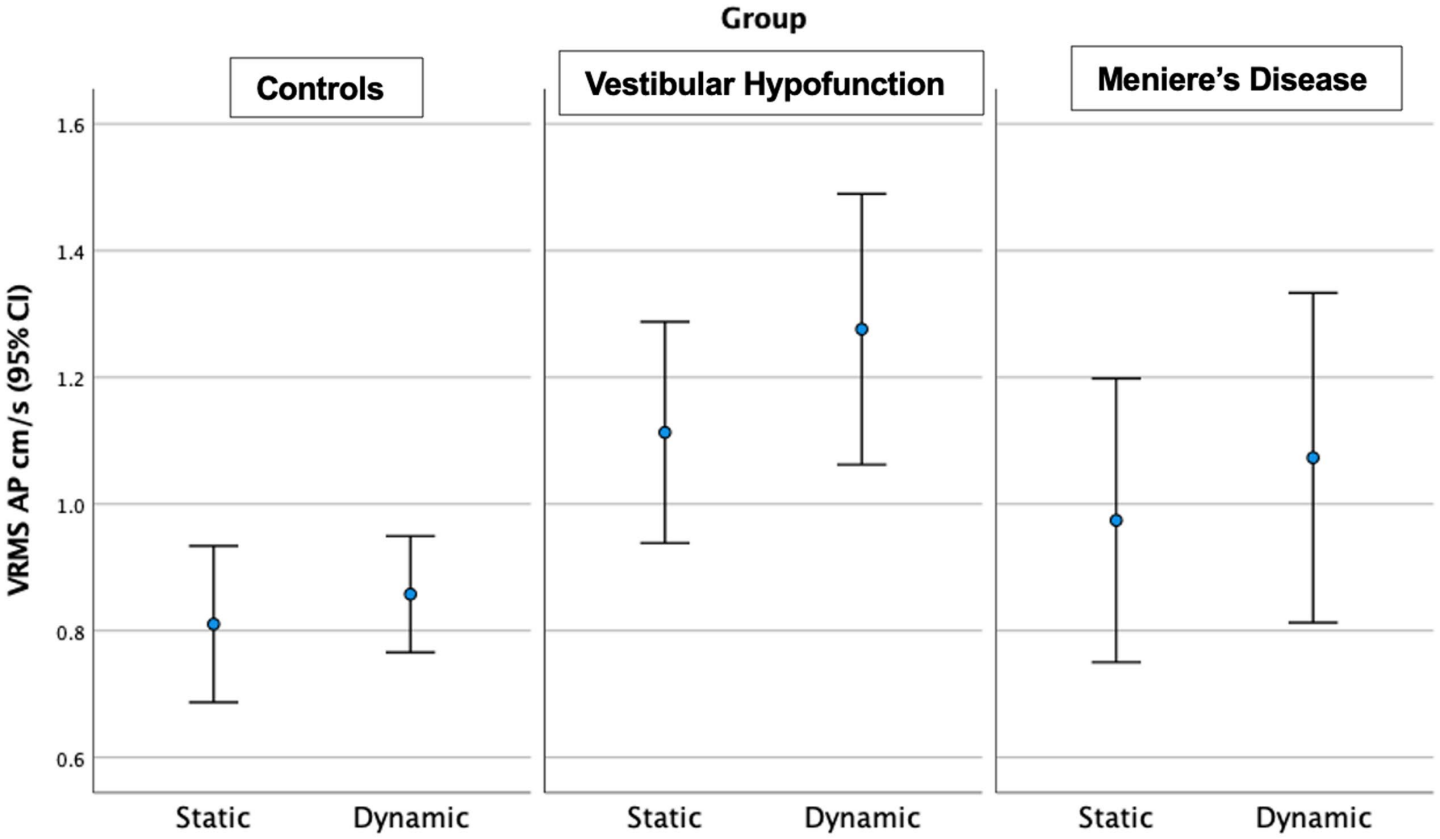
Velocity Root Mean Square (VRMS) in the anterior posterior (AP) direction in centimeters/s (y axis) for static and dynamic visual scenes (x-axis) for the three groups over 60 s. A significant increase between static and dynamic was observed for all groups (*p* < 0.001). The VH group had significantly higher VRMS AP compared to controls on both static and dynamic conditions (*p* < 0.01).

For descriptive statistics of 60 seconds performance see Table 2. We observed a significant increase in VRMS between static and dynamic visuals only in the AP direction (*p* < 0.001). See Figure 3.

**Table 2:** Descriptive statistics for 60 s analysis.

	ML static cm/s	ML dynamic cm/s	AP static cm/s	AP dynamic cm/s	Pitch static rad/s	Pitch dynamic rad/s	Yaw static rad/s	Yaw dynamic rad/s	Roll static rad/s	Roll dynamic rad/s
Control										
Mean	0.54	0.45	0.81	0.86	0.03	0.02	0.03	0.02	0.02	0.02
SD	0.37	0.20	0.33	0.25	0.02	0.01	0.03	0.01	0.01	0.01
Median	0.41	0.38	0.70	0.79	0.02	0.02	0.02	0.02	0.01	0.01
Min	0.21	0.23	0.45	0.54	0.01	0.01	0.01	0.01	0.01	0.01
Max	1.80	0.97	2.14	1.82	0.09	0.07	0.14	0.07	0.08	0.06
Vestibular hypofunction										
Mean	0.76	0.71	1.11	1.28	0.04	0.04	0.03	0.03	0.02	0.02
SD	0.50	0.34	0.48	0.60	0.03	0.03	0.03	0.02	0.02	0.01
Median	0.51	0.62	0.98	1.10	0.04	0.03	0.02	0.02	0.02	0.02
Min	0.26	0.27	0.51	0.59	0.01	0.01	0.01	0.01	0.01	0.01
Max	2.21	1.40	2.29	2.86	0.17	0.18	0.20	0.10	0.09	0.06
Meniere’s disease										
Mean	0.66	0.63	0.97	1.07	0.03	0.03	0.03	0.03	0.02	0.02
SD	0.61	0.62	0.42	0.51	0.02	0.01	0.03	0.03	0.01	0.01
Median	0.46	0.42	0.42	0.94	0.02	0.02	0.02	0.02	0.02	0.02
Min	0.23	0.29	0.35	0.47	0.01	0.02	0.01	0.01	0.01	0.01
Max	2.81	2.87	1.94	2.61	0.09	0.08	0.12	0.12	0.05	0.06

Static Scene: We observed a significant main effect of ‘group’ in the AP direction only (*p* = 0.004; Figure 3). Pairwise comparisons showed that the VH group had significantly higher AP VRMS than controls (Med 0.98 Q25, 0.8, Q75 1.3 vs. Med 0.7 cm/s Q25 0.64, Q75 0.88 cm/s, Adjusted *p* = 0.003, ES = 0.73). A difference between MD and controls (MD: Med 0.8, Q25 0.71, Q75 1.2, cm/s, *p* = 0.045, ES = 0.73) was not significant after Bonferroni correction (Adjusted *p* = 0.134). There were no significant differences between MD and VH (ES = 0.23).

**Figure 4:**
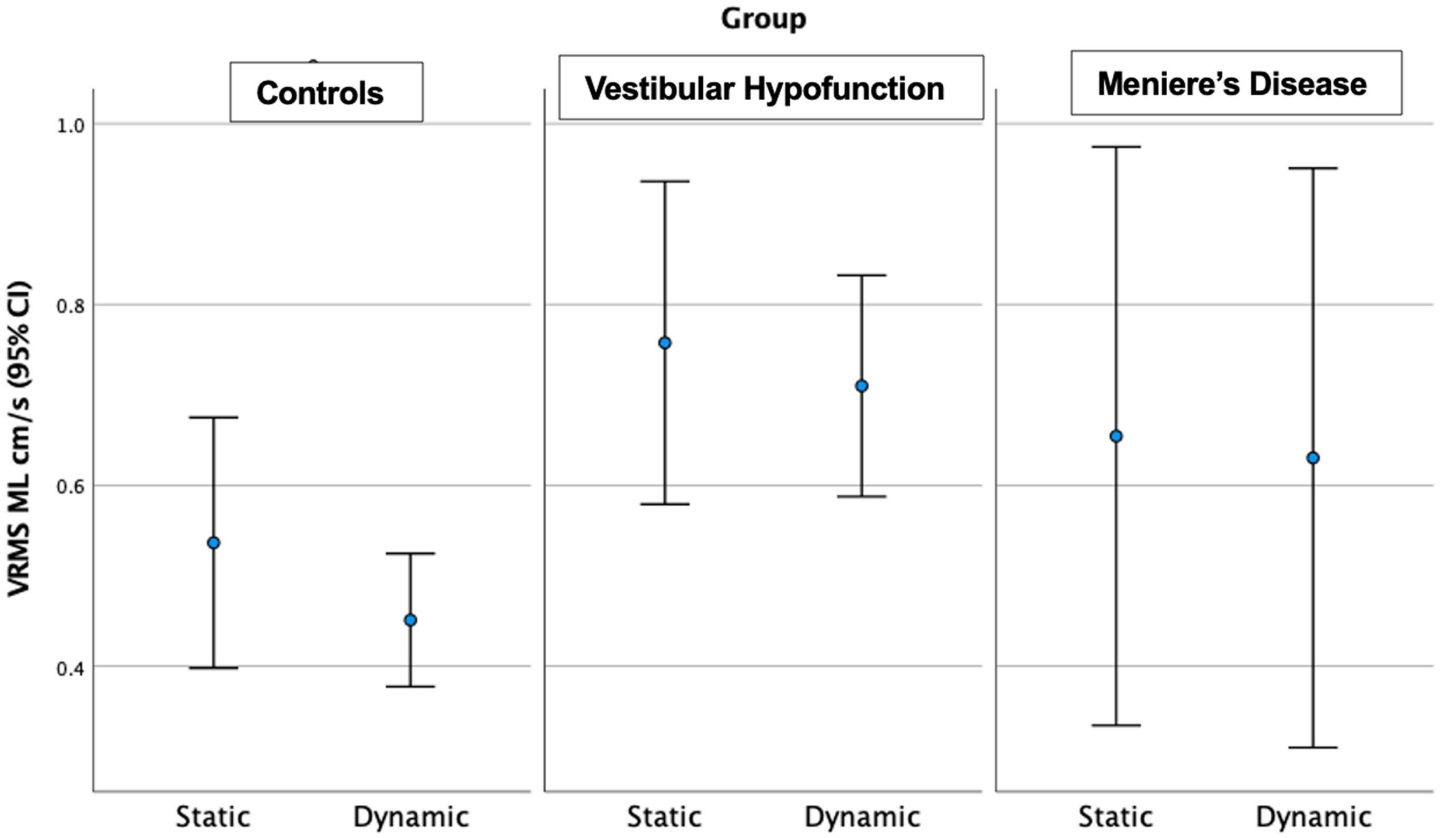
Velocity Root Mean Square (VRMS) in the medio-lateral (ML) direction in centimeters/s (y axis) for static and dynamic visual scenes (x-axis) for the three groups over 60 s. The VH group had significantly higher VRMS ML compared to controls on the dynamic condition (*p* = 0.003).

Dynamic Scene: We observed a significant main effect of ‘group’ in all directions: (ML: *p* = 0.003; AP: *p* = 0.005; Pitch: *p* = 0.015; Yaw: *p* = 0.006; Roll: *p* = 0.017; see Figure 3 for AP; Figure 4 for ML). Pairwise comparisons showed that the VH group had significantly higher VRMS than controls in all directions (ML: Med 0.62, Q25 0.45, Q75 0.99 vs. Med 0.38, Q25 0.32, Q75 0.52, cm/s, p = 0.003, ES = 0.93; AP: Med 1.1, Q25 0.85, Q75 1.52 vs. Med 0.79, Q25 0.72, Q75 1.03, cm/s, *p* = 0.004, ES = 0.91; Pitch: Med 0.025, Q25 0.021, Q75 0.050 vs. Med 0.020, Q25 0.017, Q75 0.024 rad/s, p = 0.015, ES = 0.62; Yaw: Med 0.024, Q25 0.017, Q75 0.039 vs. Med 0.017, Q25 0.013, Q75 0.022, *p* = 0.007, ES = 0.69; and Roll: Med 0.0196, Q25 0.0136, Q75 0.0261 vs. Med 0.0132, Q25 0.0105, Q75 0.0169 rad/s, *p* = 0.016, ES = 0.48). There were no significant differences between MD and VH (ES: ML = 0.18; AP = 0.36; Pitch = 0.40; Yaw = 0.25; Roll = 0.21) or MD and controls (ES: ML = 0.44; AP = 0.60; Pitch = 0.21; Yaw = 0.35; Roll = 0.24). A difference between MD and VH in ML (MD Med 0.42, Q25 0.31, Q75 0.66 cm/s, *p* = 0.045, ES = 0.18) was not significant after Bonferroni correction (Adjusted *p* = 0.135) A difference between MD and VH in yaw (MD Med 0.0177, Q25 0.0142, Q75 0.0271 rad/s, *p* = 0.047 ES = 0.25) was not significant after Bonferroni correction (Adjusted *p* = 0.14).

### 20 s analysis

**Figure 5:**
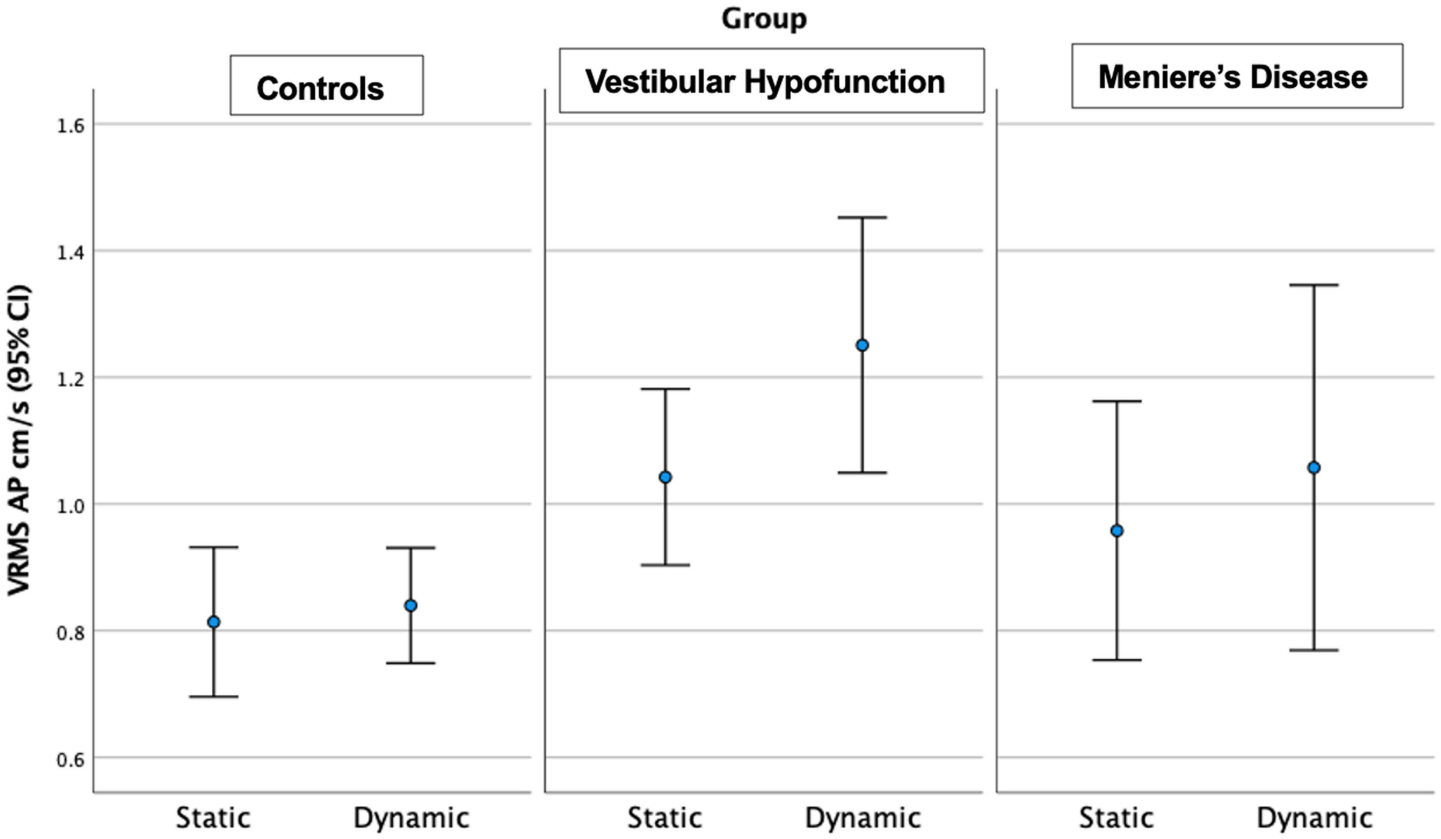
Velocity Root Mean Square (VRMS) in the anterior posterior (AP) direction in centimeters/s (y axis) for static and dynamic visual scenes (x-axis) for the three groups over the first 20 s of the scene. A significant increase between static and dynamic was observed for all groups (*p* = 0.005). The VH group had significantly higher VRMS AP compared to controls on both static and dynamic conditions (*p* < 0.05).

For descriptive statistics of 60 seconds performance see Table 3. We observed a significant increase in VRMS with dynamic visuals only in the AP direction (*p* = 0.005). See Figure 5.

**Table 3:** Descriptive statistics for 20 s analysis.

	ML static cm/s	ML dynamic cm/s	AP static cm/s	AP dynamic cm/s	Pitch static rad/s	Pitch dynamic rad/s	Yaw static rad/s	Yaw dynamic rad/s	Roll static rad/s	Roll dynamic rad/s
Control										
Mean	0.56	0.45	0.81	0.84	0.03	0.02	0.03	0.02	0.02	0.02
SD	0.42	0.22	0.32	0.24	0.02	0.02	0.04	0.01	0.02	0.01
Median	0.38	0.39	0.70	0.79	0.02	0.02	0.02	0.02	0.01	0.01
Min	0.21	0.22	0.46	0.48	0.01	0.01	0.01	0.01	0.01	0.01
Max	1.80	1.18	1.95	1.62	0.10	0.12	0.18	0.05	0.12	0.08
Vestibular hypofunction										
Mean	0.76	0.70	1.04	1.25	0.03	0.03	0.03	0.03	0.02	0.02
SD	0.55	0.33	0.39	0.56	0.02	0.02	0.04	0.02	0.01	0.01
Median	0.48	0.65	0.95	1.13	0.03	0.03	0.02	0.03	0.02	0.02
Min	0.25	0.27	0.50	0.55	0.01	0.01	0.01	0.01	0.01	0.01
Max	2.42	1.35	2.00	3.03	0.07	0.10	0.19	0.16	0.05	0.07
Meniere’s disease										
Mean	0.58	0.58	0.95	1.06	0.02	0.03	0.02	0.02	0.02	0.02
SD	0.41	0.59	0.39	0.56	0.01	0.02	0.01	0.02	0.01	0.01
Median	0.48	0.40	0.88	0.94	0.02	0.02	0.02	0.02	0.01	0.02
Min	0.24	0.27	0.39	0.54	0.01	0.01	0.01	0.01	0.01	0.01
Max	2.03	2.76	1.75	2.99	0.05	0.08	0.07	0.09	0.04	0.06

Static Scene: We observed a significant main effect of ‘group’ in the AP (*p* = 0.035) and Pitch directions (*p* = 0.02). Pairwise comparisons showed that the VH group had significantly higher AP (Med 0.95, Q25 0.75, Q75 1.27 vs. Med 0.70, Q25 0.63, Q75 0.92 cm/s, ES = 0.65, Adjusted *p* = 0.03) and Pitch VRMS (Med 0.0275, Q25 0.0195, Q75 0.0428 vs. Med 0.020, Q25 0.0154, Q75 0.0249 rad/s, ES = 0.47, Adjusted *p* = 0.02) than controls. There were no significant differences between MD and VH (ES: AP = 0.24; Pitch = 0.61) or MD and controls (ES: AP = 0.40; Pitch = 0.07).

**Figure 6:**
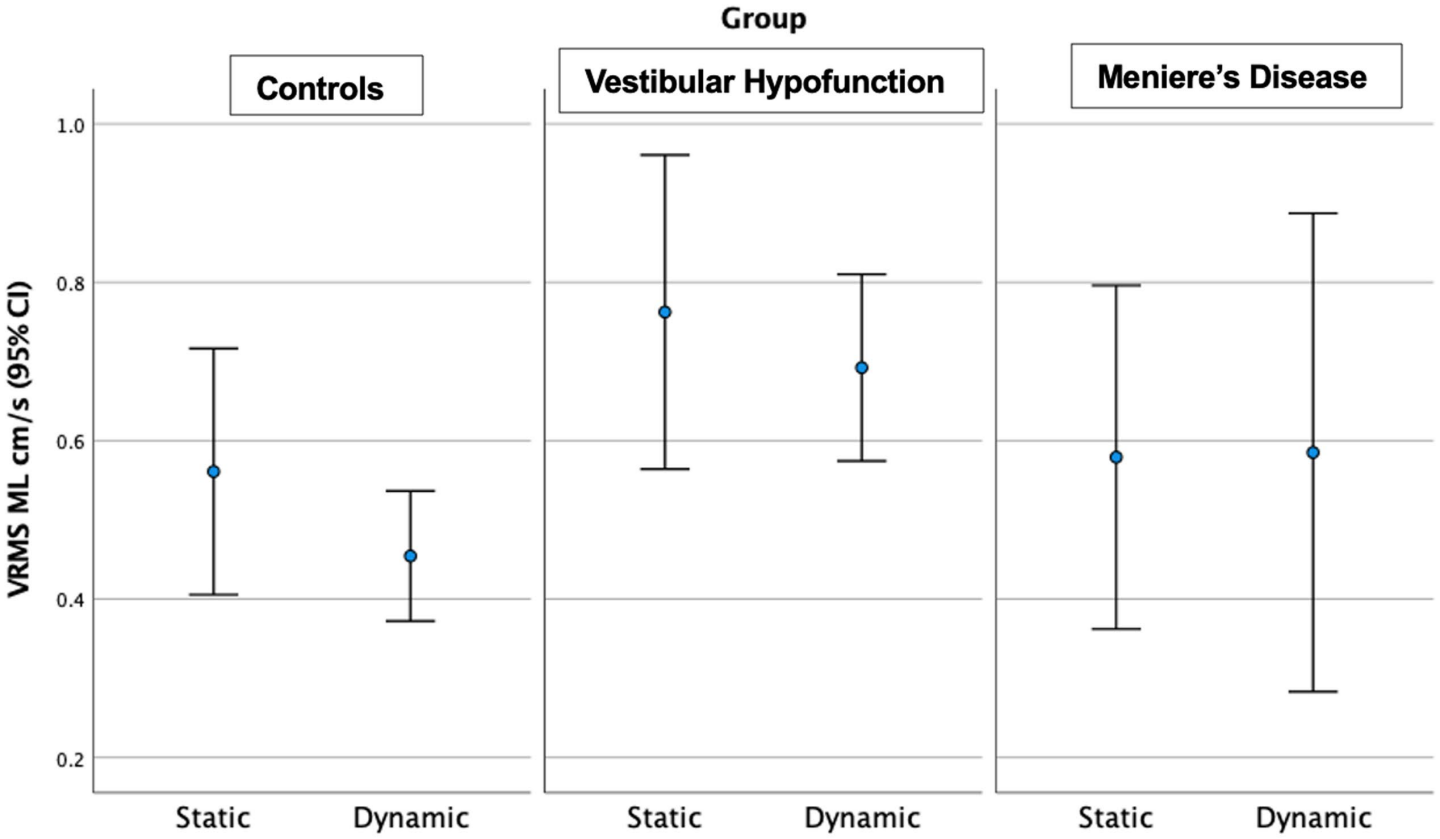
Velocity Root Mean Square (VRMS) in the medio-lateral (ML) direction in centimeters/s (y axis) for static and dynamic visual scenes (x-axis) for the three groups over the first 20 s of the scene. The VH group had significantly higher VRMS ML compared to controls on the dynamic condition (*p* = 0.006).

Dynamic Scene: We observed a significant main effect of ‘group’ in all directions: (ML: *p* = 0.004; AP: *p* = 0.002; Pitch: *p* = 0.013; Yaw: *p* = 0.008; Roll: *p* = 0.007). Pairwise comparisons showed that the VH group had significantly higher VRMS than controls in all directions: ML: Med 0.65, Q25 0.44, Q75 0.98 vs. Med 0.38, Q25 0.32, Q75 0.51 cm/s, Adjusted significance *p* = 0.006, ES = 0.85; AP: Med 1.13, Q25 0.83, Q75 1.62 vs. Med 0.79, Q25 0.66, Q75 1.04 cm/s, *p* = 0.001, ES = 0.94; Pitch: Med 0.0286, Q25 0.020, Q75 0.0389 vs. Med 0.0195, Q25 0.0177, Q75 0.0231 rad/s, *p* = 0.012, ES = 0.58; Yaw: Med 0.0244, Q25 0.0177, Q75 0.0379 vs. Med 0.017, Q25 0.0141, Q75 0.0212 rad/s, p = 0.008, ES = 0.60; and Roll: Med 0.0196, Q25 0.0138, Q75 0.0275 vs. Med 0.0133, Q25 0.0108, Q75 0.0173 rad/s, *p* = 0.005, ES = 0.48. There were no significant differences between MD and VH (ES: ML = 0.17, AP = 0.22, Pitch = 0.2, Yaw = 0.3, Roll = 0.12). VH trended toward higher ML VRMS than MD (MD Med 0.40, Q25 0.29, Q75 0.67 cm/s, *p* = 0.022, Adjusted *p* = 0.067, ES = 0.25). See Figure 6 for a 20-s comparison in the ML direction.

## Discussion

In this study, we employed a brief, HMD-based test of postural control to investigate head sway among patients with VH and MD as compared to ‘normal’ performance in healthy controls. We found significant differences in head sway between participants with VH compared to healthy controls, even when analyzing 20 s, particularly when the visual environment was dynamic. Note that the participants with VH were, on average, older than the healthy controls by 18 years. While no correlations were observed between head sway and age, age could still be a factor in these findings. Consistent with prior studies (14–16), these findings suggest that deficits in sensory integration associated with VH can be captured in a mild immersive balance assessment. Our findings that the results were similar when analyzing 60 or 20 s of the task further support the possibility for a clinical translation of this assessment. Vision influenced head sway significantly only in the AP direction, likely because the visual movement was in that direction. Despite that, the differences between groups, particularly between VH and controls were more evident with dynamic versus static visuals in all 5 directions supporting increased visual dependence in those with VH. The largest effect sizes were in the AP and ML directions, suggesting that an analysis of all five directions may not be necessary, but both static and dynamic scenes should be tested. VH is an intrinsic permanent hypofunction of the vestibular system, whereas MD is a fluctuating condition. Indeed, we observed large variability of head sway in patients with MD such that the group’s overall head sway was not significantly higher than controls but also not significantly lower than VH. Performance of the MD group appeared to be in-between the controls and VH.

Why did MD patients perform “better” than VH? Participants with MD in the current study had low SSQ before and after testing (Median = 1), ruling out the possibility that the reduction of movement was due to increased symptoms. As seen with PPPD (21), perhaps the limited head movement of MD patients in the current study was related to fear of developing symptoms (given the fluctuating nature of MD) rather than symptoms induced at time of testing, although fear was not measured directly in this study. Participants with MD displayed values that ranged from the lowest velocity among controls to the highest velocity among the VH group. This variability in head sway, also reflected in the clinical tests, such as self-reported dizziness, provides additional objective evidence for the challenges in diagnosis of MD patients, a condition known for its variability in clinical presentation (1–3). Overall, VH and MD groups were not significantly different, even though VH was higher than controls and MD was not. These results are consistent with Hong et al. that observed no significant difference between MD and VH on the Sensory Organization Test although 62% of the VH group showed abnormal vestibular ratio and only 26% of the MD (43) given the known clinical heterogeneity of MD, it is possible that a larger sample size is needed to determine if we can establish characteristic patterns of head sway for these two distinct vestibular disorders. Though all Meniere’s patients met diagnostic standards for definite MD, significant heterogeneity in symptom severity, disease progression, and individual symptomatic burden likely still exists among any MD population. None of the participants in the present study were tested during an acute Meniere’s episode. It is possible that the brevity of the test paradigm (20 s) may facilitate future recruitment of patients with active MD. While the clinical presentation and episodic nature can lead to difficulty with differential diagnosis for MD, an assessment of head sway can offer some clinical data in between MD episodes. This would allow for more portable and accessible in clinic options for differential diagnosis which do not induce symptoms, require long lengths of time, or clinical specialization (i.e., vestibular diagnostic testing—videonystagmography or vestibular evoked myogenic potentials). Our findings continue to build in the body of literature that this specific assessment is a mild perturbation which can be performed on all patients with vestibular disorders including an episodic vestibular disorder like MD.

What does the head represent in a postural response? The inverted pendulum model suggests that in quiet stance, head trajectories should correspond to movement around the ankle joint and are therefore an adequate representation of body sway (44, 45). In past work using simultaneous recording of head sway and center-of-pressure data we observed high cross correlations in young adults (46) and similar responses to visual and surface perturbations, as well as between-group differences in people with VH, people with unilateral hearing loss and healthy adults (22). Given that, it is reasonable to assume that the inverted pendulum model applies in this case as well, when participants were standing hips-width apart and were explicitly instructed to look straight ahead. Nevertheless, one must not generalize the model to other balance tasks such as dynamic movement (e.g., dodging a ball) (17), standing in a more challenging stance (e.g., tandem position) (16) or even adding an additional cognitive task that involves speaking (46). In these instances, we observed a separation between head and center-of-pressure such that the head may provide additional information but cannot use interchangeably with a force platform.

### Limitations

Our study was limited by the small sample size of the MD group, inability to test the MD group during an episode and heterogeneity of MD patients with respect to disease stage and progression. VH and MD differed from controls in demographics, specifically age and gender, although we observed no relationship between head sway and age, suggesting that age is not a likely confounding factor. It is known that anxiety and fear may influence postural control in vestibular disorders, and we did not measure either in this study.

## Conclusion

For participants with vestibular hypofunction, this work joins a growing body of literature suggesting that head sway derived from HMD is sensitive to their condition and can be clinically useful as an outcome measure to evaluate sensory integration for postural control. The assessment presented here is short, does not provoke loss of balance or symptoms and differentiates between people with VH and healthy controls even within static immersive visual environments. Increasing sensory load (here a dynamic visual environment) is associated with increased differences between groups. This work also adds to the body of literature illustrating the challenges in finding objective biomarkers to differentiate Meniere’s Disease. We observed large variability in a small MD group on head sway metrics, as well as other self-reported questionnaires. A larger sample as well as patients with worse symptoms at time of testing could elucidate whether head sway via HMD could become a viable test in this population. The similar finding between 20- and 60-s scene and the full portability of the system with an in-clinic testing setup could help these future endeavors.

## Data availability statement

The raw data supporting the conclusions of this article will be made available by the authors, without undue reservation.

## Ethics statement

The studies involving humans were approved by the Institutional Review Board of Mount Sinai and the New York University Committee on Activities Involving Human Subjects. The studies were conducted in accordance with the local legislation and institutional requirements. The participants provided their written informed consent to participate in this study.

## Author contributions

JK: Conceptualization, Writing – original draft, Writing – review & editing, Funding acquisition, Project administration, Supervision. MC: Conceptualization, Writing – review & editing, Funding acquisition, Project administration, Supervision. AL: Conceptualization, Writing – original draft, Writing – review & editing, Data curation, Formal analysis, Funding acquisition, Investigation, Methodology, Project administration, Supervision, Visualization.
